# Framing emotions: using cinema as a tool for emotional intelligence in education

**DOI:** 10.3389/fpsyg.2025.1650676

**Published:** 2025-10-13

**Authors:** Sobi Thomas, Paul Manalil

**Affiliations:** ^1^Lincoln University College, Petaling Jaya, Malaysia; ^2^Marian College Kuttikkanam Autonomous, Kuttikkanam, India

**Keywords:** emotional intelligence, educational psychology, cinema, affective learning, empathy, narrative pedagogy, reflective learning

## Introduction

In the contemporary educational landscape, emotional intelligence (EI) is increasingly recognized as a fundamental component of student development, alongside cognitive skills (Estrada et al., 2021). While curricula have traditionally prioritized intellectual competencies, affective learning—the acquisition of values, attitudes, and emotional understanding—has often remained peripheral. Emotional intelligence, defined as the ability to perceive, manage, and respond to emotions, plays a pivotal role in academic engagement, social behavior, and psychological wellbeing (Thomas and Allen, 2021). This opinion article examines the potential of cinema, particularly narrative and literary adaptations, as a tool for developing EI within educational settings (Sastre et al., 2019). Through cinematic immersion, students engage in reflective and empathetic learning processes that traditional modes of instruction may not fully facilitate.

## Cinema and emotional intelligence: theoretical underpinnings

Emotional intelligence, as articulated by Goleman and others, comprises self-awareness, self-regulation, empathy, motivation, and social skills (Tyler, 2015). Affective learning environments foster these competencies by integrating emotional content with academic instruction. Within this context, cinema provides a dynamic medium that stimulates affective and cognitive responses through storytelling, visual imagery, and character development. The concept of narrative transportation—where individuals become deeply engaged in a story—is central to cinema's educational potential (Moore and Miller, 2020). This immersive experience facilitates empathy, emotional regulation, and critical thinking, essential attributes of EI.

Added to that, cinematic teaching accommodates the concepts of experiential learning, under which learners actively interpret and emotionally process materials. By seeing characters struggle with ethical choices, interpersonal conflicts, and mental difficulties, learners are exhorted to consider their own affective frameworks and interpersonal dealings (Eweida et al., 2024). This mode of engagement facilitates the integration of the affective and cognitive domains of learning, the primary goal of educational psychology.

Recent classroom studies confirm the pedagogical value of cinema in cultivating socio-emotional learning. For example, Eweida et al. (2024) demonstrated how cinematic simulation improved nursing students' ability to regulate emotions and manage stress during psychiatric clinical training. Similarly, MacAllister (2024) showed that tragic film narratives fostered deeper ethical reasoning and intercultural sensitivity among university students. In a different context, Chico? (2024) documented how filmmaking projects enhanced adolescents' empathy and emotional communication skills. These findings provide empirical grounding to the argument that cinema can significantly contribute to the development of emotional intelligence in formal education.

## Pedagogical integration of cinema: practical reflections

Referencing the classroom environment of higher learning in communication and media studies, I have discovered how film can be used as a strong instrument of emotional sensitization. Films such as *Taare Zameen Par, The Pianist, Kireedam*, and *Schindler's List* have intricate story lines which appeal to students at the individual as well as emotional level. On one occasion, the screening of *Kireedam* ended in an unplanned discussion on mental illness, family pressure, and responsibilities toward society—topics never taught in the course outline but springing spontaneously from the filmic context.

To illustrate more concretely, I once structured a classroom session around the film *Taare Zameen Par* (Khan and Gupte, 2007), which portrays the struggles of a child with dyslexia. Before viewing, students were asked to reflect on challenges faced by children who “do not fit” into conventional schooling models. During the screening, they were encouraged to note scenes that evoked strong emotional responses. In the post-viewing discussion, students identified with the protagonist's feelings of isolation and recognized the role of supportive teachers in fostering resilience. As a follow-up activity, they prepared reflective journals linking the film's themes to their own school experiences. This exercise not only deepened empathy but also prompted critical thinking about inclusive education and emotional awareness in learning environments.

These examples illustrate film's power to engender emotional discussion and reflection and to bridge the gap from scholarly topic to lived experience. Documentaries about real events or cultural stories can also engender intercultural sensitivity and ethical reasoning and assist students in developing their emotional literacy and global orientation (MacAllister, 2024).

**Figure 1:**
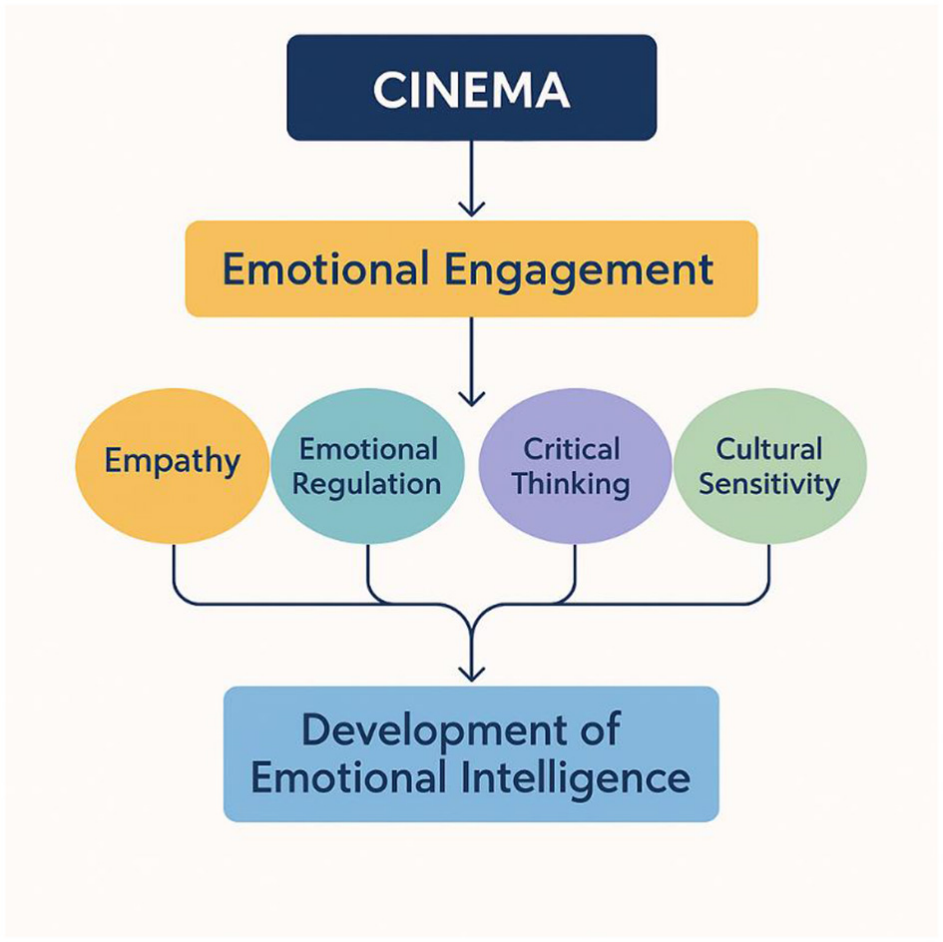
Affective learning through cinema: this conceptual flowchart illustrates how cinematic engagement fosters emotional intelligence by enhancing empathy, emotional regulation, critical thinking, and cultural sensitivity.

As depicted in the conceptual flowchart (Figure 1), cinematic engagement serves as a catalyst for developing emotional intelligence by fostering empathy, strengthening emotional regulation, promoting critical thinking, and nurturing cultural sensitivity.

## Cinematic engagement and emotional skill development

Cinema contributes to the development of emotional intelligence through several mechanisms:

1. Empathy activation: the viewer is motivated to empathize with characters, increasing their ability to adopt multiple emotional perspectives. Neuroscientific work correlates the process with mirror neuron system activation and provides a possible biological substrate for the development of empathy through narrative viewing (Pisters, 2015).
2. Emotional regulation exercise: having intense emotional scenes as part of the safe classroom setting allows for better ability to observe and process emotional responses, promoting regulation techniques as well as emotional resilience.
3. Moral and ethical reasoning: film stories sometimes pose moral issues which demand subtle reasoning. The discussion of such stories helps students acquire judgment and social responsibility (Manzanero, 2023).
4. Cultural and social sensitivity: world films provide students with perspectives on different views of the world, fostering receptivity, tolerance, and trans-cultural empathy.
5. Memory and engagement: content with an emotional appeal boosts retention and mental engagement, which is consistent with the findings from affective neuroscience correlating emotional arousal with enhanced memory encoding (Antonopoulou and Halkiopoulos, 2024). As depicted in the conceptual flowchart (Figure 1), cinematic engagement serves as a catalyst for developing emotional intelligence by fostering empathy, strengthening emotional regulation, promoting critical thinking, and nurturing cultural sensitivity.

## Framework for educational implementation

To maximize the pedagogical value of cinema in developing EI, a structured approach is essential:

- Pre-viewing orientation: introduce the film's themes, historical or cultural context, and anticipated emotional content to prepare students intellectually and affectively.
- Active viewing strategies: encourage note-taking on emotional reactions, character arcs, and pivotal narrative moments to foster mindful engagement.
- Post-viewing reflection: facilitate discussions that explore emotional responses, ethical implications, and personal connections (Kimura et al., 2019). Reflective writing or creative tasks such as rewriting scenes can deepen introspection. For instance, after screening The Pianist, students may be asked to compose short reflective essays exploring how scenes of survival and loss relate to concepts of resilience and empathy discussed in class.
- Interdisciplinary linkages: connect film content to psychological theories, cultural studies, and ethical frameworks to support comprehensive understanding.
- Assessment tools: employ qualitative evaluation methods such as emotional self-assessments, reflective essays, and peer discussions to gauge emotional learning outcomes.

## Discussion

Despite its promise, the use of cinema in emotionally oriented education requires careful consideration. Not all students may be ready to process intense emotional content. Educators must be attuned to the diverse emotional backgrounds of learners, offering trigger warnings and support mechanisms when necessary (Neathery, 2024). Classroom discussions should be conducted within an emotionally safe space, with clear norms for respectful and non-judgmental dialogue (Cebula et al., 2022).

While cinema offers unique opportunities for affective learning, its integration is not without challenges. Students' emotional readiness varies, and some may experience discomfort when engaging with intense or traumatic content. Additionally, films are interpreted differently across cultural backgrounds, which may lead to divergent classroom responses (Uhrig et al., 2016). Another limitation is the difficulty of isolating cinema's impact on emotional intelligence, since outcomes are often influenced by broader social and personal factors (Zwiky et al., 2024). To address these concerns, educators can provide contextual framing, use trigger warnings, and facilitate structured dialogue that respects diverse perspectives. Such strategies help ensure that cinematic pedagogy remains both emotionally safe and educationally productive.

Assessment remains a challenge, as emotional growth is difficult to quantify. Nonetheless, narrative evaluations, reflective journals, and student-led presentations can offer insight into students' emotional development (Nazari et al., 2024).

The COVID-19 pandemic has underscored the urgency of integrating emotional support into education. Students have faced unprecedented disruptions, social isolation, and mental health challenges. In this context, emotionally resonant teaching methods are more vital than ever (Gogoi et al., 2022). Cinema can serve as both a reflective and therapeutic tool, enabling students to process complex emotions and reconnect with shared human experiences.

In online and hybrid learning environments, short films, digital storytelling, and student-created video projects can extend the benefits of cinematic engagement. These formats offer flexibility and accessibility, making emotional learning feasible across varied learning contexts (Phan et al., 2024).

## Conclusion: toward emotionally intelligent pedagogy

As educational paradigms shift toward holistic student development, the integration of emotional intelligence becomes imperative. Cinema, with its capacity for narrative immersion and emotional evocation, offers a compelling modality for emotional pedagogy (Chico?, 2024). It supports the development of empathy, emotional regulation, critical thinking, and intercultural awareness—skills essential for navigating the complexities of contemporary life.

Educational institutions should support film-integrated learning through curriculum design, educator training, and interdisciplinary collaboration. Further research is needed to empirically examine the long-term impact of cinematic pedagogy on emotional and academic outcomes. As we seek to cultivate emotionally intelligent learners, cinema stands as a transformative educational ally.
